# Nursing students’ knowledge and attitudes on sexually transmitted infections prevention at training institution in Namibia

**DOI:** 10.4102/hsag.v29i0.2483

**Published:** 2024-02-16

**Authors:** Lonia U. Hamunyela, Hileni N. Niikondo, Monika N. Nakweenda

**Affiliations:** 1 School of Nursing and Public Health, Faculty of Health Sciences and Veterinary Medicine, University of Namibia, Windhoek, Namibia

**Keywords:** knowledge, attitudes, sexually transmitted, infections, student nurses

## Abstract

**Background:**

Insufficient knowledge about sexually transmitted infections (STIs) among nursing students can impact patient care and outcomes. To address this, comprehensive STI education is crucial.

**Aim:**

This study aimed to assess knowledge and attitudes of nursing students regarding STI prevention and control.

**Setting:**

The research was conducted at a nursing training institution in Windhoek, Namibia.

**Methods:**

The research employed a cross-sectional design with 73 participants.

**Results:**

Outcomes revealed that 63.0% had satisfactory knowledge and 79.5% exhibited positive attitudes. A significant association was noted between knowledge and participants’ sex. Male participants displayed poor knowledge (70%). No significant relationship existed between demographic characteristics and attitudes.

**Conclusion:**

The study concluded lower than expected knowledge regarding STIs among nursing students that implicated patients’ care.

**Contribution:**

Integrating STI education into nursing curricula can improve students’ competences that enhance patients’ care.

## Introduction

Untreated, sexually transmitted infections (STIs) can have serious complications on pregnancy outcome, such as stillbirths, neonatal death, low birth weight and prematurity sepsis, pneumonia, neonatal conjunctivitis and congenital deformities. Sexually transmitted infections can also lead to cervical cancer, pelvic inflammatory disease and infertility in women (Shivute et al. 2019; WHO 2021).

In lower-income countries, STIs remain the main public problem contributing to morbidity and mortality as well as increased financial costs. Cases of STIs have been high since 2014 (CDC 2018). Tanzania recorded syphilis as one of the most common STIs, which contributed to 51% of stillbirths, 24% of preterm live births and 17% of all adverse pregnancy outcomes (CDC 2018). Early diagnosis and treatment are crucial, to prevent complications imposed by late diagnosis. Despite the implementation of preventive measures that include abstaining from sex, condom use, vaccination, health education and male circumcision, the prevalence of STIs remains a concern.

Namibia reported an increased 12.7% prevalence of chlamydia and gonorrhoea among persons aged between 15 and 49 years (Shivute et al. 2019). Consequently, the latest report on Namibia human immunodeficiency virus (HIV) prevalence was higher (19.16%) among women compared to men (13.46%) (U.S. Department of State 2022). The overall reported rate of chlamydial conjunctivitis in infants was relatively stable during 2014–2018, ranging from 1.4 to 2.3 cases per 100 000 live births. Similarly, the rate of gonococcal conjunctivitis in infants remained relatively constant and low during 2014–2018, ranging from 0.2 to 0.4 cases per 100 000 live births (CDC 2018).

The knowledge of STIs among healthcare workers aimed at reducing the complications of STIs is vital for the management of patients. Meanwhile, stakeholders also advocated a continuum of STI services to the public as a health strategic approach (Shivute et al. 2019).

### Nurses’ knowledge on sexually transmitted infections

One-third of students from third-world societies were reported unfamiliar with any STIs, other than acquired immunodeficiency syndrome (AIDS) (World Health Organization [WHO] 2019). According to a study in Italy, students had insufficient knowledge on the prevention of STIs with some indicating that birth control pills can also prevent infections (Visalli et al. 2019). It was further revealed that most students lacked sufficient knowledge on the complications of STIs (Subotic et al. 2022).

According to a study in Kenya, about 38% of the surveyed nurses lack knowledge of proper identification and diagnosis of genital ulcer diseases, whereas about 45% depended on laboratory findings to make a diagnosis (Mumbi et al. 2020). In addition, it was found that nursing students and graduate nurses had inadequate knowledge on AIDS management (Ngcobo & Mchunu 2019). Moreover, insufficient knowledge is attributed to a lack of appropriate training on syndromic management of STIs (Mumbi et al. 2020).

### Nurses’ attitudes on sexually transmitted infections

Most studies alluded that having good knowledge may influence good attitudes towards patients with STIs (Alahdal, Basingab & Alotaibi 2020; Mutaru et al. 2023). However, knowledge do not always lead to positive attitudes (Boakye & Mavhandu-Mudzusi 2019). Negative attitudes may not be a favourable platform for patients seeking treatment on STIs, as it may deter patients from reporting symptoms because of the fear of stigma (WHO 2021). Therefore, student nurses in their primary years of the profession should be expected to display the right attitudes as a good health promotion tool to encourage patients, to fully report their symptoms.

### Problem statement

Sexually transmitted infections continue to increase in Namibia despite the adoption of the WHO global health sector strategy on STIs (WHO 2021). Although the guideline is introduced in the healthcare facilities, its application is not fully integrated within the nursing training curriculum. In addition, little is known regarding the knowledge and attitudes of nursing students in relation to the prevention and control of STIs. However, nursing students in the second year of study are allocated to health facilities and are expected to manage patients presenting STI symptoms. Insufficient knowledge and negative attitudes among nursing students regarding STIs can have a detrimental impact on patient care (Boakye & Mavhandu-Mudzusi 2019). Therefore, lack of evidence on students’ knowledge and attitudes prompted the researchers to conduct the study among the undergraduate nursing students at a training institution in Namibia.

### Aim

The aim of the study was to determine the knowledge and attitudes of undergraduate nursing students towards STI prevention and control.

The specific objectives of the study were:

- to determine the knowledge and attitudes of undergraduate nursing students towards STI prevention and control;
- to describe the knowledge and attitudes of undergraduate nursing students towards STI prevention and control;
- to analyse the relationship between the demographic characteristics of undergraduate nursing students and their level of knowledge and attitudes towards STI prevention and control.

## Research design and methods

### Study design

The study utilised a quantitative, cross-sectional and analytical approach to determine and describe the knowledge and attitudes of nursing students at the University of Namibia (UNAM), towards the prevention and control of STIs. The demographic characteristics of students were compared with knowledge and attitudes variables to determine the relationship.

### Study setting

This study was conducted at a training institution where undergraduate nursing students received theoretical teaching to prepare them for clinical practice. The students integrated their knowledge into practice during biweekly clinical allocation. Prior to this allocation, the students underwent orientation on theoretical knowledge, which included the basics of STIs. Because some students were assigned to primary healthcare centres involved in screening and diagnosing patients with STIs, the knowledge and attitudes of these students were of utmost importance.

### Population and sampling strategy

The study targeted a specific population comprising 109 second-year students enrolled in the Bachelor of Nursing Science program at the main campus during the year 2019. The convenience sampling method was employed to determine a minimum sample size of 86 participants, calculated using the Yamane’s formula with a confidence interval of 95% and a margin of error of 5% (*n* = *N*/1 + *Ne*^2). However, because of availability and willingness to participate, only 73 students (84.88% response rate) were included in the study.

### Data collection instrument

A self-reported questionnaire with closed-ended questions and a Likert scale ranging between agree, disagree and neutral was developed in English, which is the formal medium of instruction in Namibia. The questionnaire was divided into three main domains, which were demographic characteristics of participants, knowledge on STIs and attitudes towards STI prevention and control.

The validity of the instrument was ensured with a pilot study on five students who were further excluded from the main study to avoid bias. Overall, the pilot study suggested that no major changes in structure or content of the questionnaire were necessary. Moreover, the instrument underwent an expert’s thorough assessment to ensure its face validity. Furthermore, content validity was ensured by including STIs’ information from literature, which represents the full domain of the content related to the nursing care of patients. The internal consistency of the instrument was ensured by a Cronbach’s alpha value of 0.043, thus making the data collection instrument reliable.

### Data collection procedure

After approval of the proposal by the institution, the principal investigator sought consent from participants. Questionnaires were distributed by the principal author to second-year nursing students during their free period while on campus and collected after completion on the same day. The questionnaire took approximately 10 min to complete. A locked cabinet and encrypted computer were used to store the data.

### Data analysis

The collected raw data were entered into SPSS version 27.0 for analysis. To assess knowledge levels, each correct response was assigned a score of 5, while an incorrect response received a score of 0. The knowledge items consisted of 10 questions, resulting in a total score range of 0–50. The knowledge scores were then categorised into three groups: poor (score ≤ 30), satisfactory (score between 31 and 40) and good (score between 41 and 50).

Similarly, for attitudes, a score of 5 was assigned to ‘agree’ responses, a score of 3 to ‘neutral’ responses and a score of 0 to ‘disagree’ responses. The attitudes section comprised 14 items, giving a total score range of 0–70. The attitudes scores were further categorised into three groups: negative (score ≤ 30), neutral (score between 31 and 50) and positive (score between 51 and 70).

Descriptive analysis was conducted to provide an overview of the knowledge and attitudes of second-year nursing students regarding STI prevention and control. This involved reporting the frequencies of sociodemographic characteristics and the proportions of knowledge and attitudes. Inferential statistics were performed using Fisher’s exact test to examine any possible associations between sociodemographic characteristics as independent variables and knowledge and attitudes as dependent variables. Because of small, expected cell counts and violations of the assumptions of chi-square, Fisher’s exact test was used. A *p*-value of ≤ 0.05 was considered statistically significant to reject the null hypothesis. The results were presented using tables and graphs, and narrative reporting was utilised to interpret the findings.

### Ethical considerations

The principal researcher obtained an ethical clearance certificate on 16 July 2019, from the School of Nursing Ethical Committee (ethical clearance reference number: SON/013/2019) to conduct the research for partial fulfilment of the Bachelor of Nursing, Clinical Honours. Participants provided informed written consent after a comprehensive explanation of the study’s objectives, ensuring voluntary participation without any coercion in accordance with the 1964 Helsinki Declaration. To maintain confidentiality and anonymity, participants were not required to disclose their names on the questionnaire. Additionally, measures were taken to prevent harm, with well-structured questions designed to avoid distress. To ensure justice, equal opportunity was provided to all participants and a standardised questionnaire was utilised to maintain consistency in data collection.

## Results

A total of 73 participants provided demographic information including age, gender, marital status and religion. Participants were also asked to disclose if they had previously attended any STIs training and whether they had been enrolled as nurses before pursuing their bachelor’s degree.

Table 1 provides an overview of the 73 participants in the study. Most participants were female students, accounting for 63 (86.3%) of the sample, while the remaining 10 (13.7%) were male students. The participants’ main age range was between 19 years and 24 years, with 62 (91.8%) falling within this age group. Christian believers constituted the majority, with 70 (95.9%) participants. Additionally, 49 (67.1%) respondents had not attended any STI training, and 65 (89.0%) had not been enrolled as nurses before their current bachelor’s degree programme.

**TABLE 1:** Demographic characteristics of respondents (*N* = 73).

Demographic characteristics	Frequency (*n*)	Percentage (%)
**Sex**		
Female	63	86.3
Male	10	13.7
**Marital status**		
Married	6	8.2
Single	67	91.8
**Age group (years)**		
19–24	62	84.9
25–29	4	5.5
30–35	7	9.6
**Religion**		
Christian	70	95.9
Nonbeliever	1	1.4
Other	2	2.7
**Attended STI prevention and control training**		
Yes	24	32.9
No	49	67.1
**Enrolled nurse before**		
Yes	8	11.0
No	65	89.0

*Source:* Abrahams, F.R., Daniels, E.R., Niikondo, H.N. & Amakali, K., 2023, ‘Students’ knowledge, attitude and practices towards pressure ulcer prevention and management’, *Health SA Gesondheid* 28(0), a2180. https://doi.org/10.4102/hsag.v28i0.2180

STI, sexually transmitted infections.

Table 2 displays the frequencies on the knowledge of respondents on STIs. The findings revealed that most of the respondents (71, 97.3%) were aware that abstinence reduces the risk of acquiring STIs. Similarly, 64 (87.7%) respondents recognised that circumcision could decrease the chances of infection. However, a significant number of respondents (63, 86.3%) were unaware of any vaccine that can protect against hepatitis B, an STI. Furthermore, 53 (72.6%) of the respondents did not know that it is possible to contract STIs without being sexually active. In terms of STIs in pregnancy, 53 (72.6%) of the respondents were unaware of the negative effects it can have on an unborn baby.

**TABLE 2:** Frequencies on the nursing students’ knowledge of sexually transmitted infections (*N* = 73).

Knowledge statements	Yes		No	
	*n*	%	*n*	%
Circumcision decreases the risk of infections.	64	87.7	9	12.3
Someone can get STIs even though she or he is not sexually active.	20	27.4	53	72.6
STIs in pregnancy has negative effects on unborn baby.	20	27.4	53	72.6
Gonorrhoea is a treatable and curable STIs.	41	56.2	32	43.8
HPV can lead to cancer in women.	54	74.0	19	26.0
A woman can tell by the way her body feels if she has a sexually transmitted disease.	51	69.9	22	30.1
A person who has genital herpes must have open sores to give the infection to his or her partner.	33	45.0	40	54.8
There is a vaccine that can protect a person from getting hepatitis B.	63	86.3	10	10.7
STIs does not increase the risk of HIV.	17	23.3	56	76.0
Abstinence lowers the risk of getting STIs.	71	97.3	2	2.7

*Source:* Abrahams, F.R., Daniels, E.R., Niikondo, H.N. & Amakali, K., 2023, ‘Students’ knowledge, attitude and practices towards pressure ulcer prevention and management’, *Health SA Gesondheid* 28(0), a2180. https://doi.org/10.4102/hsag.v28i0.2180

STI, sexually transmitted infections; HPV, human papillomavirus.

Table 3 indicates that most respondents, 66 (90%), expressed positive agreement regarding partner treatment for women diagnosed with STIs. Similarly, 66 (90.4%) of the respondents believed that young people should receive information about STIs. However, a group of 37 (50.7%) respondents were unsure if individuals can be infected with STIs without exhibiting any symptoms. Regarding common symptoms of STIs, 49 (67.1%) of the respondents disagreed that abnormal discharge from genital areas and itching were indicative of such infections. Furthermore, a total of 42 (53.4%) respondents expressed disagreement with the notion that STIs can be transmitted through skin-to-skin contact or by kissing an individual with a herpes blister.

**TABLE 3:** Frequencies on the nursing students’ attitudes on sexually transmitted infections (*N* = 73).

Attitudes statements	Agree		Disagree		Neutral	
	*n*	%	*n*	%	*n*	%
Some people go for STIs screening even though they were not sick.	48	65.8	17	23.3	8	11.0
Effective use of condoms can prevent transmission of STIs.	65	89.0	6	8.2	2	2.7
Some people can have STIs without any symptom.	36	49.3	24	32.9	13	17.8
STIs can be treated or cured.	62	84.9	4	5.5	7	9.6
If a woman is diagnosed with STIs, is it needed for her partner to be treated also?	66	90.0	4	5.5	3	4.1
STIs can lead to cancer.	45	61.6	12	16.4	16	21.9
Abnormal discharge from genital part and itching were not the common symptom of STIs.	30	17.8	49	67.1	11	15.1
STIs can be spread through skin-to-skin contact and simply kissing someone with a herpes blister.	34	46.6	23	31.5	16	21.9
Maintaining personal and environmental hygiene will reduce the risk of getting infections.	47	64.4	12	16.4	14	19.2
Isolating an individual who had STIs can help prevent spread of the disease.	8	11.0	51	69.9	14	19.2
Young people should get information/knowledge about STIs.	66	90.4	0	0.0	7	9.6
After graduation you will not hesitate to work on a unit that has a high number of people living with HIV.	44	60.3	14	19.2	15	20.5
A nurse should keep distance during the care of HIV/STI patients.	9	12.3	63	86.3	1	1.4
A body of a patient with AIDS should not be touched without wearing gloves.	13	17.8	51	69.9	8	11.0

*Source:* Abrahams, F.R., Daniels, E.R., Niikondo, H.N. & Amakali, K., 2023, ‘Students’ knowledge, attitude and practices towards pressure ulcer prevention and management’, *Health SA Gesondheid* 28(0), a2180. https://doi.org/10.4102/hsag.v28i0.2180

STI, sexually transmitted infections.

### Association between demographic characteristics and knowledge of respondents (*N* = 73)

Students’ knowledge was compared to their demographic characteristics, using Fisher’s exact test. The results indicated that Christians had higher knowledge regarding the hepatitis B vaccine, with 62 (88.6%) of the respondents being aware of its preventive effect against active infection, while respondents of other religious beliefs had no knowledge (*p* = 0.046) (Table 4). Respondents’ demographic characteristics were also compared to an overall knowledge score. An overall knowledge score indicated that 26 (35.6%) of the respondents had poor knowledge, while 46 (63.0%) had satisfactory knowledge, and only 1 (1.4%) demonstrated good knowledge (Table 5). A statistically significant association was observed between the variable sex and the overall knowledge score, as 7 of the 10 male respondents (70%) had poor knowledge of STIs compared to 19 of the 63 female respondents (30.2%) who had poor knowledge of STIs (*p* = 0.045) (Table 5).

**TABLE 4:** Association between demographic characteristics of respondents and their attitudes (*N* = 73).

Statement	Variables	Group	Agree		Disagree		Neutral		*χ* ^2^	*P*
			*n*	%	*n*	%	*n*	%		
Do you believe that STI can be treated or cured?	Religion	Christians	61	83.6	3	4.1	6	8.2	11.525	0.044
		Nonbeliever	0	0.0	1	1.4	0	0.0		
		Other	1	1.4	0	0.0	1	1.4		
Do you believe maintaining personal and environmental hygiene will reduce the risk of getting infections?	Sex	Female	44	60.3	10	13.7	9	12.3	7.251	0.020
		Male	3	4.1	2	2.7	5	6.8		
After graduation you will not hesitate to work on a unit that has a high number of HIV-positive patients.		Female	40	54.8	13	17.8	10	13.7	5.134	0.050
		Male	4	5.5	1	1.4	5	6.8		
Do you believe isolating an individual who had STI can help prevent spread of the disease?	Being an enrolled nurse	Enrolled nurse	1	1.4	3	4.1	4	5.5	5.398	0.039
		Not enrolled nurse	7	9.6	48	65.7	10	13.7		

*Source:* Abrahams, F.R., Daniels, E.R., Niikondo, H.N. & Amakali, K., 2023, ‘Students’ knowledge, attitude and practices towards pressure ulcer prevention and management’, *Health SA Gesondheid* 28(0), a2180. https://doi.org/10.4102/hsag.v28i0.2180

STI, sexually transmitted infections.

**TABLE 5:** Frequencies of the overall level of knowledge and attitudes of respondents (*N* = 73).

Variables	Overall knowledge and attitudes levels													
	Knowledge							Attitudes						
	Poor		Satisfactory		Good		*p*	Negative		Neutral		Positive		*p*
	*n*	%	*n*	%	*n*	%		*n*	%	*n*	%	*n*	%	
**Sex**							0.045							1.000
Female	19	30.2	43	68.3	1	1.6		1	1.6	12	19.0	50	79.4	
Male	7	70.0	3	30.0	0	0.0		0	0.0	2	20.0	8	80.0	
**Marital status**							1.000							0.159
Married	2	33.3	4	66.7	0	0.0		0	0.0	3	50.0	3	50.0	
Single	24	35.8	42	62.7	1	1.5		1	1.5	11	16.4	55	82.1	
**Age group (years)**							0.903							0.297
19–24	22	35.5	39	62.9	1	1.6		1	1.6	10	16.1	51	82.3	
25–29	1	25.0	3	75.0	0	0.0		0	0.0	1	25.0	3	75.0	
30–35	3	42.9	4	57.1	0	0.0		0	0.0	3	42.9	4	57.1	
**Religion**							0.163							0.132
Christian	24	34.3	45	64.3	1	1.4		1	1.4	12	17.1	57	81.4	
Nonbeliever	0	0.0	1	100	0	0.0		0	0.0	1	100	0	0.0	
Other	2	100	0	0.0	0	0.0		0	0.0	1	50.0	1	50.0	
**Attended STI prevention and control training**							1.000							1.000
Yes	9	37.5	15	62.5	0	0.0		0	0.0	5	20.8	19	79.2	
No	17	34.7	31	63.3	1	2.0		1	2.0	9	18.4	39	79.6	
**Enrolled nurse before**							1.000							0.270
Yes	3	37.5	5	62.5	0	0.0		0	0.0	3	37.5	5	62.5	
No	23	35.4	41	63.1	1	1.5		1	1.5	11	16.9	53	81.5	

**Total**	**26**	**35.6**	**46**	**63.0**	**1**	**1.4**		**1**	**1.4**	**14**	**19.2**	**58**	**79.5**	

*Source:* Abrahams, F.R., Daniels, E.R., Niikondo, H.N. & Amakali, K., 2023, ‘Students’ knowledge, attitude and practices towards pressure ulcer prevention and management’, *Health SA Gesondheid* 28(0), a2180. https://doi.org/10.4102/hsag.v28i0.2180

STI, sexually transmitted infections.

Significance of Fishers’ exact test (*p* ≤ 0.05).

### Association between demographic characteristics and attitudes of respondents (*N* = 73)

Equally, students’ attitudes were compared to their demographic characteristics, using Fisher’s exact test. The results indicated that Christian believers demonstrated positive attitudes towards STIs, as the majority 61 (83.6%) believed that STIs can be cured (*p* = 0.04). Regarding hygiene, 44 female respondents (60.3%) believed that personal hygiene may decrease the risk of STIs (*p* = 0.02). However, 40 (54.8%) female respondents expressed agreement in working with HIV patients after graduation (*p* ≤ 0.05) (Table 4). Respondents who were not previously enrolled nurses (48, 65.7%) appropriately did not believe that isolating a person who is infected with STIs can prevent the spread of the disease (*p* = 0.04).

Respondents’ demographic characteristics were compared to an overall attitudes score. The results indicated that only 1 (1.4%) of respondents had negative attitudes, 14 (19.2%) had a neutral attitude and 58 (79.5%) had positive attitudes. However, no statistically significant association was observed regarding the overall attitudes score and the respondents’ demographic characteristics (sex, *p* = 1.000; marital status, *p* = 0.159; age group, *p* = 0.297; religion, *p* = 0.132; attended STI training, *p* = 1.000 and enrolled nurse, *p* = 0.270) (Table 5).

## Discussion

The aim of the study was to determine the knowledge and attitudes of nursing students regarding the control and prevention of STIs. In this study, most respondents were young, similar to the studies that were reviewed, such as the 22-year median age of medical students at one medical school in the Southeast of England (Arthur et al. 2021). Most studies among young students and learners revealed adequate knowledge on the transmission and prevention of STIs (Nyasulu et al. 2018; Petry et al. 2019). This was confirmed in this study that was specifically carried out among nursing students who showed to have satisfactory knowledge on some STI concepts. The findings revealed a high proportion of female participants, comprising 63 individuals (86.3%), which is similar to the results reported in a Canadian study, where 38 respondents (76%) displayed similar outcome on the knowledge, and beliefs about human papillomavirus vaccination (Wilson et al. 2021).

The proportion of students who had not been enrolled as nurses before their current bachelor’s degree program was high (65, 89.0%) as compared to students who were previously enrolled nurses (8, 11.0%). The findings are different from a study done by Jadoon et al. (2022) who found low proportion of the bachelor’s degree students in a Pakistan study about students’ knowledge on STIs (57, 29.2%).

Furthermore, 97.3% of respondents agreed that abstinence lowers the risk of getting STIs. The same findings as the latter were reported in Italy where many students were aware that abstinence significantly lowers the risk of STI transmission (Cegolon et al. 2022). The knowledge concept was also validated by an Indian study (Small, Kim & Yu 2021). However, a few questions had unexpected wrong answers whereby some nursing students did not know that someone can get STIs without being sexually active, and that STIs in pregnancy has negative effects on the unborn baby. The lack of knowledge regarding STIs among nursing students in Africa is common but worrisome, as they are expected to give health education and manage STI-related conditions (Badawi et al. 2019; Mansor, Ahmad & Rahman 2020). It is therefore necessary to include the content of STI prevention and control in the curriculum as from the first year of nursing study to reduce the risks to the clients (Pete et al. 2019).

The attributes of positive attitudes towards patients with STIs among the respondents were consistent with most of the recent findings of nurses’ attitudes towards patients with STIs (Boakye & Mavhandu-Mudzusi 2019; Leyva-Moral et al. 2020). The positive attitudes are attributed to good knowledge, as there is a co-relationship between knowledge and attitudes (Boakye & Mavhandu-Mudzusi 2019). Other literature also reported that knowledge could determine nurses’ discriminatory or unethical attitudes towards patients living with the STIs, mainly HIV (Boakye & Mavhandu-Mudzusi 2019). Consequently, if students have adequate knowledge, then they are likely to have positive attitudes. Nonetheless, most students could not agree whether some people can have STIs without any symptoms. The negative attitudes may lead the nurses to the refusal of screening asymptomatic STI clients. Similarly, most students disagreed on the question whether abnormal discharge from genital areas and itching were common symptoms of STIs. This can lead to failure of correctly diagnosing STIs among patients required for proper treatment (Small, Kim & Yu 2021).

On comparison, a statistically significant association was found between religion and knowledge; Christians knew about the availability of a vaccine that protect people from getting hepatitis B infection (Fisher’s exact test, *p* = 0.046). Good knowledge among Christian students on STIs was also reported by others (Badawi et al. 2019).

Similarly, student responses were cross-tabulated to test whether there was an association between their demographic characteristics and their attitudes. Female nursing students believed that maintaining personal and environmental hygiene will reduce the risk of getting infections, unlike other students in other studies, who believed that contracting the virus is by supernatural means (Badawi et al. 2019). Furthermore, female students indicated that they did not hesitate to work in a unit that has a high number of people living with HIV. On a positive note, students who were Christians believed that STIs can be treated or cured, indicating caring attitudes. Similarly, students who were not enrolled nurses previously correctly believed that isolating patients infected with STIs does not prevent the spread of the disease compared to students who were enrolled nurses previously (*p* = 0.039). It is a concern if students who had previous nursing experience display prejudice towards patients with STIs. In comparison, the study by Tsadik, Lam and Hadush (2019) reported that prejudgment attitudes of nurses may reduce the health-seeking behaviour among patients, thus contributing to the spread of STIs.

## Limitations

The findings of this study provided strong information on STIs, especially on the attitudes of second-year nursing students at the UNAM. However, the findings were limited by the fact that the population was small, and therefore the findings cannot be generalised for all nursing students in Namibia. The self-reported data collection instrument might prejudice the study to possible invalid answers from respondents. Furthermore, the cross-tabulation analysis was done with Fisher’s exact test as the assumptions of chi-square were violated.

## Conclusion

The study revealed that, overall, students did not have good knowledge as expected but had satisfactory knowledge (63.0%) about the prevention and control of STIs. However, there was an unequal distribution of knowledge among students, with some respondents having misconceptions about STI transmission. Non-Christian believers were found to lack knowledge about the hepatitis B vaccine, and a concerning 70.0% of male respondents exhibited poor knowledge. Only a small percentage (1.4%) of respondents demonstrated good knowledge. By implication, nursing students were found in a weaker position in terms of knowledge to care for patients and clients diagnosed with STIs.

The study findings indicated an overall positive attitude (79.5%) among respondents. Positive attitudes among students may result in reduced STIs because students can raise awareness towards the prevention of STIs in the society. However, 20.5% of respondents who portray negative attitudes may contribute negatively towards the prevention of STIs in the country.

It is crucial that all nursing students acquire basic knowledge about STI prevention. Additionally, it is important for all nursing students to display positive attitudes towards patients without judgement.

The study recommends that the training curriculum for nursing students at the initial level includes comprehensive information about STIs to improve knowledge and shape attitudes. Further research with a larger sample size among a similar population is suggested to enhance the generalisability of findings. Moreover, a different approach should be explored to investigate the perceptions and experiences of nursing students when working with patients diagnosed with STIs.
